# Multiple Lung Metastases of Papillary Thyroid Carcinoma Detected by Detailed Pathological Examination: A Case Series

**DOI:** 10.7759/cureus.80112

**Published:** 2025-03-05

**Authors:** Ryusei Yoshino, Nanami Ujiie, Shunsuke Yasuda, Masahiro Kitada

**Affiliations:** 1 Thoracic Surgery and Breast Surgery, Asahikawa Medical University Hospital, Asahikawa, JPN; 2 Thoracic Surgery and Breast Surgery, Asahikawa Medical University Hospital, Asahikwa, JPN

**Keywords:** malignant thyroid tumor, papillary thyroid carcinoma, pulmonary metastasis, surgery, thyroglobulin

## Abstract

Papillary thyroid carcinoma (PTC) is the most common histological type of malignant thyroid tumor, and the lungs are one of the most frequent sites of distant metastasis. Although the progression of the disease remains slow even after the appearance of pulmonary metastases, and there are reports of long-term survival cases, no clear criteria have been established for performing aggressive biopsies of metastatic lesions.

The subject was a 79-year-old male who was detected to have left cervical lymphadenopathy of unknown origin six years back. After further examination, cervical lymph node metastasis of PTC was suspected. Five years ago, the patient underwent total thyroidectomy and left cervical lymph node dissection. Two years ago, a nodule was observed in the right upper lobe of the lung, but it was managed with observation. A chest computed tomography scan revealed an irregular nodule measuring 15×14 mm in the S1 segment of the right upper lobe. Under suspicion of primary lung cancer, it was decided to perform intraoperative rapid diagnosis, and if malignant, a right upper lobectomy with mediastinal lymph node dissection would be carried out. Intraoperative rapid diagnosis confirmed malignancy, and a right upper lobectomy and mediastinal lymph node dissection were performed. Histopathological examination revealed findings of papillary adenocarcinoma, leading to a diagnosis of secondary lung cancer (pT1cN0cM0, pStage IA3). In addition, findings suggestive of multiple metastases from PTC were also observed. Similarly, no adjuvant therapy was administered for the PTC lung metastases, and a policy of careful observation was adopted.

This case is a valuable report of pulmonary metastasis from PTC discovered through detailed histopathological examination of the resected lung. Furthermore, performing aggressive surgical biopsy and detailed histopathological examination to establish a diagnosis is worth considering from the perspective of personalized medical care for each patient.

## Introduction

Papillary thyroid carcinoma (PTC) is the most common variety of thyroid cancer (70%-80% of cases) and the most common endocrine neoplasia. Classically, PTC has been classified as a well-differentiated thyroid cancer, together with follicular thyroid cancer, because of its good prognosis and low mortality rate. In some cases, distant metastases can occur, with the lungs being one of the most frequent sites of metastasis [1, 2].

The clinical course of PTC patients with pulmonary metastases is highly variable, ranging from asymptomatic micro-metastases to extensive pulmonary lesions with symptoms. Notably, when pulmonary metastases are diagnosed early and treated appropriately, long-term survival can often be achieved. However, advanced pulmonary metastases may serve as a prognostic factor for poor outcomes. On the other hand, there are also reports of cases with slow progression and long-term survival spanning decades, even after the appearance of pulmonary metastases [1, 3].

Indicators that suggest the possibility of metastases include serum thyroglobulin levels, chest computed tomography (CT), and fluorodeoxyglucose positron emission tomography (FDG-PET) scans [4]. However, due to the diverse outcomes in PTC patients with pulmonary metastases, no clear criteria have been established for performing aggressive biopsies of metastatic lesions.

As with the present case, reports of PTC pulmonary metastases identified through detailed histopathological examination of resected lung tissue remain limited. This report describes the clinical course of such a case in detail. Furthermore, a discussion is provided by comparing this case with four additional cases from our experience.

## Case presentation

The patient was a 79-year-old male with a height of 174 cm, a weight of 65 kg, and a BMI of 21.5 kg/m². He had a smoking history of two packs per day for 55 years, with a Brinkman Index of 1100. His medical history included a total thyroidectomy performed five years earlier for papillary thyroid carcinoma (PTC) with known comorbidities of chronic obstructive pulmonary disease and hypertension.

Six years ago, the patient was found to have left cervical lymphadenopathy of unknown origin. Subsequent investigations revealed suspected cervical lymph node metastasis of PTC. Five years ago, the patient underwent total thyroidectomy and left cervical lymph node dissection. Histopathological findings revealed multiple papillary thyroid microcarcinomas (pT1a [m], pEx0, pN1b 4/10, pStage IVA) with negative surgical margins. The patient was treated with radioiodine therapy (Iodine-131).

Two years ago, a nodule in the right upper lobe of the lung was identified, which was followed up with chest CT scans. Compared to two years prior, an increase in nodule density was observed, prompting a transbronchial biopsy; however, no definitive diagnosis was made. CYFRA, CEA, SLX, ProGRP, and NSE are all considered tumor markers, specifically used in the diagnosis and monitoring of lung cancer, with each indicating the presence of different cancer cell types: (a) CYFRA (Cytokeratin 19 Fragment): Primarily associated with squamous cell carcinoma of the lung. (b) CEA (Carcinoembryonic Antigen): Can be elevated in various cancers, including colon and lung adenocarcinoma. (c) SLX (Sialyl Lewis-X antigen): A marker that can be elevated in various cancers, including lung cancer. (d) ProGRP (Pro-gastrin releasing peptide): Considered a highly sensitive marker for small cell lung carcinoma (SCLC). (e) NSE (Neuron-Specific Enolase): Also associated with SCLC and can be used to monitor its progression.

**Figure 1:**
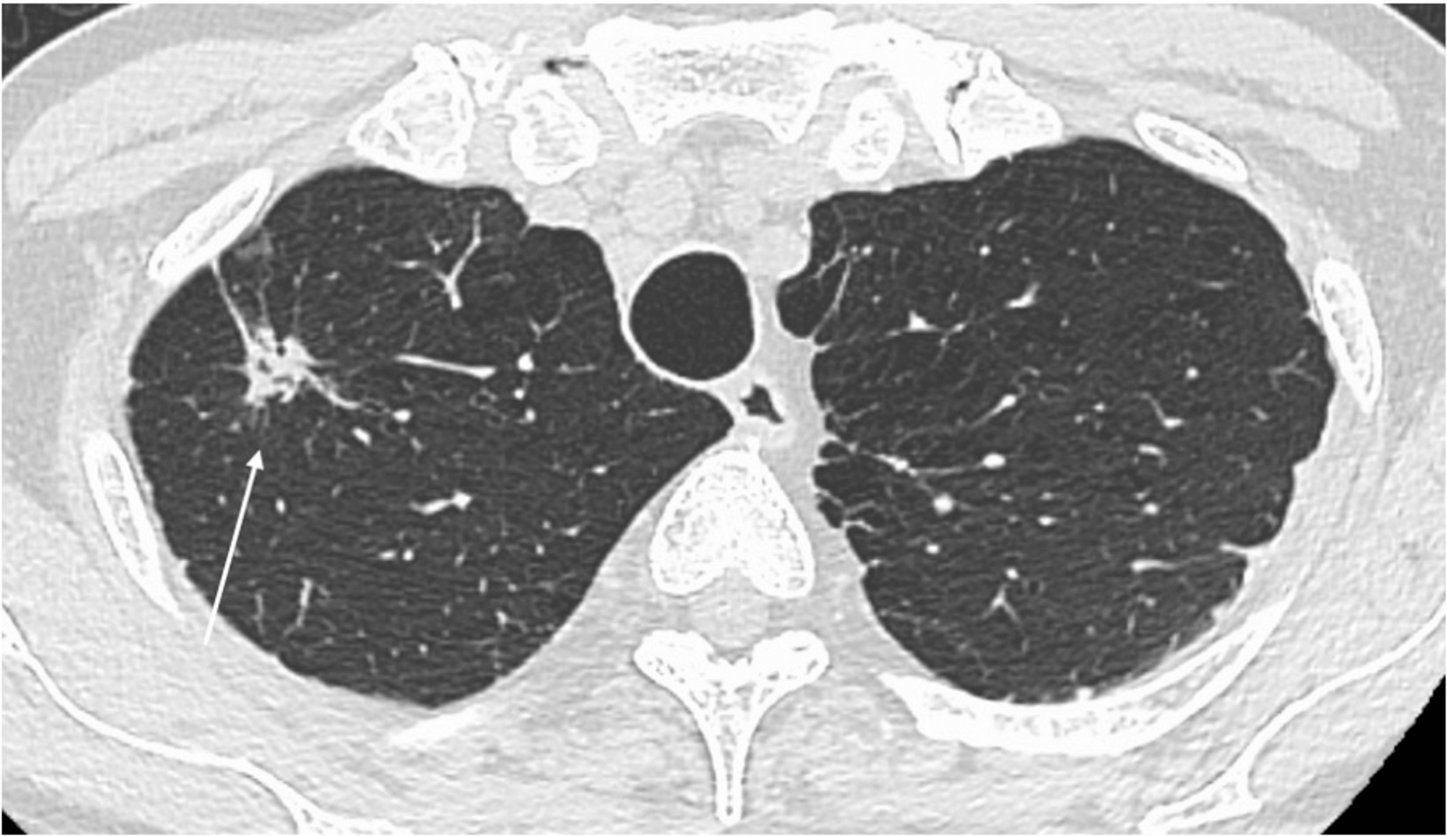
Chest computed tomography (CT) findings (axial). An irregular nodule 15 x 14 mm in size was seen in the S1 upper lobe of the right lung (arrow).

Tumor markers (CYFRA, CEA, SLX, ProGRP, NSE) were within normal limits. Serum thyroglobulin levels showed a gradual increase over time, with a preoperative value of 47.7 ng/mL. Chest X-rays showed no abnormalities, while chest CT scans revealed an irregular nodule measuring 15×14 mm in the S1 segment of the right upper lobe (Figure 1). No hilar lymphadenopathy was detected. FDG-PET was not performed. Pulmonary function and electrocardiogram tests showed no abnormalities. Based on these findings, the patient was suspected of having primary lung cancer. A surgical plan was made to perform intraoperative rapid diagnosis, followed by right upper lobectomy and mediastinal lymph node dissection if malignancy was confirmed.

The surgery was performed under general anesthesia in the left lateral decubitus position with video-assisted thoracoscopy. The tumor was partially resected using an automatic stapler and submitted for rapid diagnosis. The diagnosis of adenocarcinoma was confirmed, and a right upper lobectomy with mediastinal lymph node dissection (ND-1b) was performed. The operation lasted 2 hours and 30 minutes. The postoperative course was uneventful; the chest drain was removed on postoperative day 2, and the patient was discharged on postoperative day 7.

**Figure 2:**
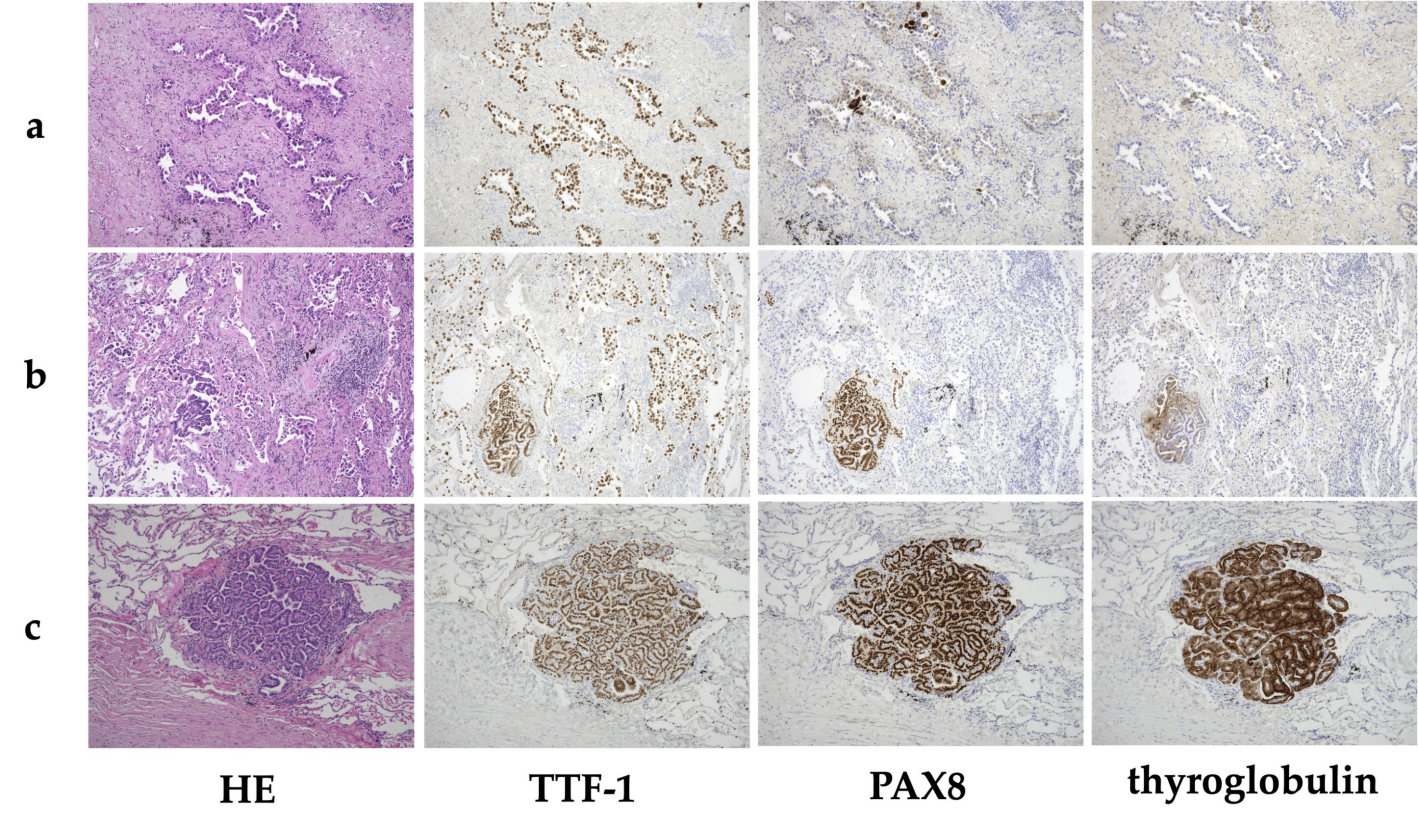
Gross and histopathological findings. a: Hematoxylin-eosin (HE) staining revealed papillary adenocarcinoma with atypical cells with chromatin-stained nuclei of unequal size and eosinophilic cytoplasm, and papillary, adenoblastic, and micropapillary growth. Immunohistochemistry was positive for thyroid transcription factor 1 (TTF-1), negative for paired box 8 (PAX8), and negative for thyroglobulin (×10), b: There were scattered papillary growths of tumor cells with nuclear grooves, nuclear inclusion bodies, and frosted nuclei in the vicinity of the primary lung cancer. Immunohistochemistry was positive for TTF-1, PAX8, and thyroglobulin (×10), c: Similar findings were seen in a resected specimen of the upper lobe of the right lung, suggesting multiple metastases of papillary thyroid carcinoma (×10).

The histopathological examination findings are as follows. The partial resection specimen submitted for rapid diagnosis revealed atypical cells with hyperchromatic nuclei of variable sizes and eosinophilic cytoplasm, proliferating in papillary, acinar, micropapillary, and lepidic patterns. The findings were consistent with papillary adenocarcinoma (papillary 60%, acinar 20%, micropapillary 10%, lepidic 10%; WHO Grade 2), leading to a diagnosis of secondary lung cancer (pT1cN0cM0, pStage IA3). Immunohistochemistry showed TTF-1 positivity, PAX8 negativity, and thyroglobulin negativity (Figure 2a). The surgical margins were negative, with no vascular or lymphatic invasion. Additionally, scattered clusters of tumor cells with nuclear grooves, intranuclear inclusions, and ground-glass nuclei, proliferating in a papillary pattern, were observed near the secondary lung cancer. Immunohistochemistry showed thyroid transcription factor 1 (TTF-1)** **positivity, paired box 8 (PAX8)positivity, and thyroglobulin positivity (Figure 2b). Similar findings were observed in the resected right upper lobe specimen (Figure 2c), suggesting multiple pulmonary metastases from PTC.

After consultation with the patient, it was decided not to administer postoperative adjuvant therapy for secondary lung cancer. Similarly, no adjuvant therapy was provided for the pulmonary metastases from PTC, and a strategy of careful observation was adopted. Six months post-surgery, there was no evidence of recurrence.

## Discussion

PTC is typically a tumor with a favorable prognosis, with cervical lymph node metastasis being the most common type of spread. However, distant metastases have been reported in 5-10% of cases, with the lungs being the most common metastatic site [5]. Pulmonary metastases from PTC are often asymptomatic and are usually incidentally detected through imaging studies. The typical imaging findings include ground-glass opacities or micro-nodules, and in advanced cases, solid masses may be observed [6]. Diagnostic modalities such as radioactive iodine scintigraphy, serum thyroglobulin measurement, and CT are useful for identifying pulmonary metastases. Serum thyroglobulin levels, in particular, are considered a key marker for evaluating the presence of lung metastases and assessing therapeutic efficacy in PTC patients after total thyroidectomy [1]. However, differentiating pulmonary metastases of PTC from primary lung cancer can be challenging, often requiring immunohistochemical analysis. Generally, PTC is characterized by positivity for thyroglobulin and PAX8, while primary lung cancer shows positivity for TTF-1 and negativity for thyroglobulin [7]. In this case, immunohistochemistry also played a pivotal role in establishing the diagnosis.

This case, in which pulmonary metastases of PTC were incidentally discovered through pathological examination, underscores the importance of always considering metastases when serum thyroglobulin levels are elevated or micro-nodules are observed in the lungs. Surgical biopsy of pulmonary metastases in PTC patients is considered uncommon. While advanced thyroid cancer is typically associated with a poor prognosis, some small-scale studies have reported cases where patients remained stable for decades [1, 8]. Furthermore, radiologists often interpret multiple small pulmonary nodules as inflammatory changes. For patients with distant metastases, treatment options include surgery, external radiation therapy, and iodine-131 ablation [9]. Therefore, early diagnosis is crucial for determining individualized treatment strategies, including careful observation. In this case, the failure to perform FDG-PET imaging when serum thyroglobulin levels began to rise represents a point for reflection. During follow-up of PTC patients, any increase in serum thyroglobulin levels or the presence of pulmonary micro-nodules should always prompt consideration of metastases.

Furthermore, surgical resection of pulmonary micro-nodules, followed by detailed pathological examination, can provide significant diagnostic value. In this case, surgical resection was performed due to suspected secondary lung cancer. However, even without a primary lung cancer lesion, surgical biopsy of pulmonary nodules would have been valuable for diagnostic purposes. Although there is no large-scale study linking surgical biopsy of pulmonary metastases in PTC to improved survival outcomes due to the rarity of the disease [1, 8], cases have been reported where patients remained stable for decades with no additional treatments beyond thyroid-stimulating hormone (TSH)suppression. Thyroid hormone suppression therapy is designed to lower serum TSH levels using doses of thyroid hormone in excess of what would normally be required to maintain a euthyroid state. The basis of this therapy is the knowledge that TSH is a growth factor for thyroid cancer, so that lower serum TSH levels might be associated with decreased disease activity. This suggests that diagnostic biopsy holds importance. Aggressive consideration of surgical biopsy for pulmonary micro-nodules in PTC patients should remain a priority, and further accumulation of similar cases is anticipated. This case, which led to a diagnosis based on detailed thin-slice pathological examination and close collaboration with pathologists, highlights the importance of multidisciplinary communication.

Among the cases of pulmonary resection performed at our institution, four, including this one, were diagnosed as pulmonary metastases of PTC based on postoperative histopathological findings (Table 1). The median age was 69.5 years (range: 58-82 years). Serum thyroglobulin levels were elevated preoperatively in all cases. In three of the four cases, pulmonary nodules were detected on preoperative chest CT scans. Three cases had a history of thyroid cancer, while one was diagnosed with primary PTC following pulmonary resection. The recurrence-free interval was relatively long, and among the three cases where pulmonary metastases were surgically resected, no evident recurrence or additional metastases were observed. Although the number of cases is small, in patients with a history of thyroid cancer, the presence of pulmonary nodules or rising serum thyroglobulin levels should prompt consideration of metastases, and resection of pulmonary metastatic lesions should be considered. Given the scarcity of reports on surgical resection for pulmonary metastases, this case contributes valuable data to the literature.

**Table 1:** Clinicopathological features of pulmonary metastasis cases of papillary thyroid carcinoma (PTC) experienced in our department. Cases 1 to 4 have not been published previously. Case 4 is the case described in this paper. *A partial pneumonectomy is a surgical procedure that removes one or more lobes of a lung. It's a type of lung resection, which is surgery to remove a part or all of a lung.

Case	Age	Sex	Thyroglobulin antibodies (<28.0 IU/ml)	Thyroglobulin (<33.7 ng/ml)	Computed tomography	Location of lung metastasis	Lung area with nodules	Number of lung metastases	Tumor size (mm)	Technique	Time to recurrence (months)	Postoperative therapy	Duration of disease-free recurrence to date (months)
1	82	Female	<10	55.7	nodule	Right	S10	1	9	Partial Pneumonectomy*	272	No treatment and observation	61
2	58	Female	12	39.8	nodule	Left	S8	5 or more	7	Partial Pneumonectomy*	0	Radiation therapy after total thyroidectomy	30
3	60	Female	13.4	50.7	nodule	Right	S6	1	18	Lung Lobectomy	448	No treatment and observation	29
4	79	Male	14.6	47.7	micronodule	Right	S1	1	2	Lung Lobectomy	60	Died after 6 months of no treatment and follow-up	-

## Conclusions

This case report describes a case of lung metastasis that was diagnosed by an increase in serum thyroglobulin levels and the presence of pulmonary micro-nodules after surgery for papillary thyroid cancer. Although this disease is generally considered to have a favorable prognosis, performing aggressive surgical biopsy and detailed histopathological and immunohistochemical examinations to establish a diagnosis is worthwhile from the perspective of personalized medicine for each patient. We hope that this case will contribute to further research.
